# Synopsis of the Course of Lectures on Materia Medica

**Published:** 1855-11

**Authors:** 


Art. VII.—*Synopsis of the Course of Lectures on Materia Medica and Pharmacy, delivered in the University of Pennsylvania.* By Joseph Carson, M. D. Second edition; revised. Philadelphia: Blanchard & Lea. 1855. pp. 196. 8vo.

---

The above work is an outline of the lectures on Materia Medica and Pharmacy, delivered by Dr. Joseph Carson, to the classes assembled in the University of Pennsylvania. It is intended as a guide to the leading facts and principles discussed by Dr. Carson in his course.

It is well known to students that the text books which treat of the same department pursue neither the order nor the method a lopted in the lectures to which they listen; and that much loss of time is entailed by reading all that is relevant or irrelevant to the portion of subject under consideration at any given period. The range of these works usually placed in the hands of the student is so extensive that he finds it impossible to do more than glance at them during the college terms. And hence the advantages that must accrue from assistance in the form of the present work. So long as the present brief and crowded system of Medical education obtains—so long as lecture terms consist of six lectures daily during a period of only four months—so long as diplomas are given after two years' study—just so long are works of the class we are now handling requisite to anything like fair understanding and reasonable appropriation of what is communicated by oral instruction.

The reader of this notice will please not be misled by the fact that the Synopsis was prepared for the classes of the University of Pennsylvania. It epitomizes the subject of which it treats in a very thorough and satisfactory manner, and will prove a valuable hand-book to the student in attendance upon any of the Medical Colleges in this country. D. W. Y.

